# A neurophysiologically interpretable deep neural network predicts complex movement components from brain activity

**DOI:** 10.1038/s41598-022-05079-0

**Published:** 2022-01-20

**Authors:** Neelesh Kumar, Konstantinos P. Michmizos

**Affiliations:** Computational Brain Lab, Department of Computer Science, Rutgers University, Piscataway, NJ USA

**Keywords:** Computer science, Brain-machine interface

## Abstract

The effective decoding of movement from non-invasive electroencephalography (EEG) is essential for informing several therapeutic interventions, from neurorehabilitation robots to neural prosthetics. Deep neural networks are most suitable for decoding real-time data but their use in EEG is hindered by the gross classes of motor tasks in the currently available datasets, which are solvable even with network architectures that do not require specialized design considerations. Moreover, the weak association with the underlying neurophysiology limits the generalizability of modern networks for EEG inference. Here, we present a neurophysiologically interpretable 3-dimensional convolutional neural network (3D-CNN) that captured the spatiotemporal dependencies in brain areas that get co-activated during movement. The 3D-CNN received topography-preserving EEG inputs, and predicted complex components of hand movements performed on a plane using a back-drivable rehabilitation robot, namely (a) the reaction time (RT) for responding to stimulus (slow or fast), (b) the mode of movement (active or passive, depending on whether there was an assistive force provided by the apparatus), and (c) the orthogonal directions of the movement (left, right, up, or down). We validated the 3D-CNN on a new dataset that we acquired from an in-house motor experiment, where it achieved average leave-one-subject-out test accuracies of 79.81%, 81.23%, and 82.00% for RT, active vs. passive, and direction classifications, respectively. Our proposed method outperformed the modern 2D-CNN architecture by a range of 1.1% to 6.74% depending on the classification task. Further, we identified the EEG sensors and time segments crucial to the classification decisions of the network, which aligned well with the current neurophysiological knowledge on brain activity in motor planning and execution tasks. Our results demonstrate the importance of biological relevance in networks for an accurate decoding of EEG, suggesting that the real-time classification of other complex brain activities may now be within our reach.

## Introduction

The knowledge of how the brain encodes movement components has driven the development of brain computer interfaces for several tasks, from controlling neural prostheses^1^, to targeting motor learning^2–5^. For over more than half a century, several studies have identified specialized neurons in the motor cortex as well as other cortical and subcortical areas, that encode movement components, including intent and timing^6^, direction^7^, amplitude^8^, force^9^ and speed^10^. These individual neurons are best recorded using brain implants that have sufficient spatial and temporal resolution to decode components of complex movements^11–13^. With the real-world applicability of single neuron recordings being rather limited and a subject of medical concerns and portability issues, electroencephalography (EEG) has long been offering a convenient non-invasive recording of the brain activity in real-time. However, the EEG’s main caveats, namely its low spatial resolution and ill-defined source localization, hinder its reliable decoding, despite progress in both statistical and the most recent machine learning methods^14,15^.

Traditionally, EEG decoding has relied on statistical methods that compute hand-crafted features such as common spatial pattern (CSP)^16^ and employ classifiers such as support vector machines (SVM) to segragate those features^17,18^. Statistical EEG decoding methods suffer from two fundamental limitations that impede their use in accurate real-time applications^2^. Specifically, the widely-used method, CSP, requires domain-specific hand-crafted features that need to be computed offline. CSP is also sensitive to noise and outliers in the data, which results in poor generalization^19,20^. This partly explains why CSP has achieved high accuracies on classification tasks for highly discriminatory signals, such as the classification of right- versus left-hand movement^16,21^, that decrease significantly when classifying more difficult tasks, including movement directions^22^. It is the unique ability of deep learning in addressing the main issues present in conventional statistical methods that has recently spurred the wide use of this disruptive technology in several tasks^23^.

Indeed, deep neural networks (DNN) eliminate the need for domain-specific processing through unsupervised feature learning and exhibit powerful generalization ^24^, but their applicability in decoding movement using EEG is still rather limited due to three main reasons. First, the vast majority of the efforts to employ DNN for decoding EEG use previously acquired datasets ^25^, which limits their scope to classifying gross movement components. In fact, many recent DNN works on EEG classification focus on tasks such as motion/no-motion, hand/feet, and grasping/lifting movements ^21,26,27^. Second, the combination of well-separated classes with the excessive discrimination power of DNN partly explains why the current approaches typically over-simplify EEG input representations as a 2-dimensional matrix of stacked channels vs. time data ^21^. Although such representations offer easier processing, they are also susceptible to removing spatial dependencies that exist among brain areas^28^, neglecting a significant EEG component that can be used to further improve decoding accuracy. Third, the black-box nature of the DNN methods masks the correspondence between the learned features and the underlying neurophysiology. This makes network interpretation difficult and limits its reliability for deployment in real-world applications^29^. For these reasons, an accurate decoding of complex movement components in real-time will remain out of reach without an interpretable deep network with topography-preserving EEG input representations.

**Figure 1:**
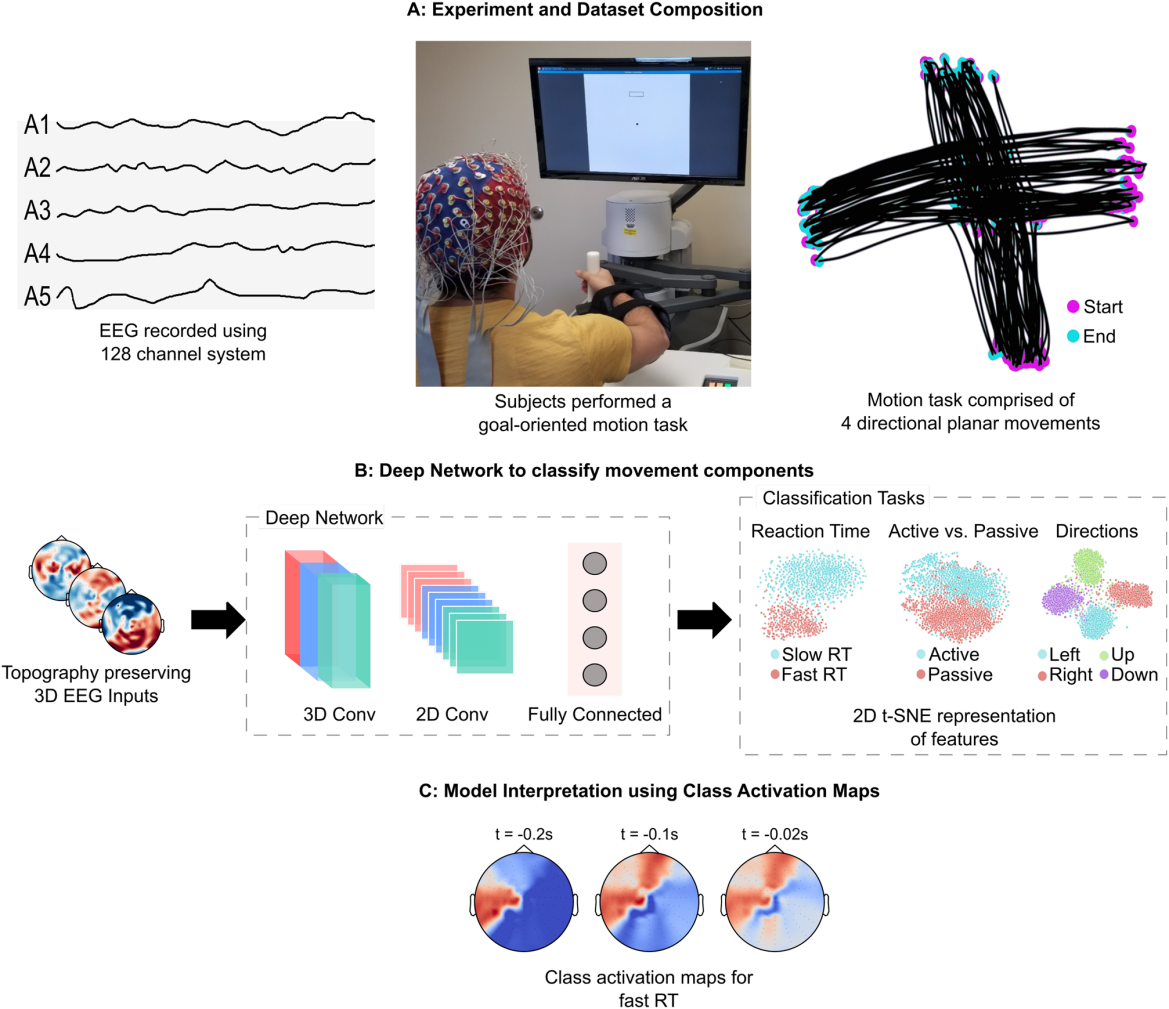
Experimental setup and study workflow. (**A**) 12 subjects performed a goal-oriented motion task (center) while high density EEG data (left) were simultaneously acquired with movement kinematics (right). (**B**) The proposed 3D-CNN received topography-preserving EEG inputs, and classified complex components of hand movements. (**C**) Gradient-weighted Class Activation Maps were developed to identify the relevant location and timing of activated brain areas per classification task.

Here, we present a neurophysiologically interpretable 3-dimensional deep convolutional neural network (3D-CNN) that captured the spatiotemporal dependencies in the EEG related to co-activated brain areas during movement. The 3D-CNN predicted complex components of hand movements performed on a plane using a back-drivable rehabilitation robot, namely (i) the reaction time (RT) for responding to the stimulus (2 classes: slow and fast), (ii) the mode of movement (2 classes: active or passive, depending on whether there was an assistive force provided by the apparatus), and (iii) the orthogonal directions of the movement (4 classes: left, right, up, and down) (Fig. 1B). The network received topography-preserving EEG inputs and performed 2D convolution in the sensor space and 1D convolution in the temporal space to extract task-discriminative spatiotemporal features. We validated the 3D-CNN on a new dataset acquired from an IRB-approved in-house experiment (Fig. 1A) using the leave-one-subject-out technique, and report average test accuracies of 79.81%, 81.23%, and 82.00% for RT, active vs. passive, and direction classifications respectively. Our proposed method outperformed the modern 2D-CNN architecture by a range of 1.1% to 6.74% depending on the classification task. The 3D input representation allowed us to interpret the 3D-CNN using the Gradient-weighted Class Activation Maps (Grad-CAM) ^30^, and suggest the EEG sensors and time segments crucial to the classification decision of the network (Fig. 1C). The identified locations and timing of the underlying brain activity aligned with the activity reported in the primary motor cortex, the pre-motor cortex, the supplementary motor area, and orbitofrontal cortex that have long been implicated in motor planning ^31^ and execution tasks^32,33^. Our results demonstrate the importance of biological relevance in 3D-CNN for accurately decoding complex movement components from EEG, suggesting that our 3D-CNN could be further studied in classifying other complex brain activities in real-time.

**Figure 2:**
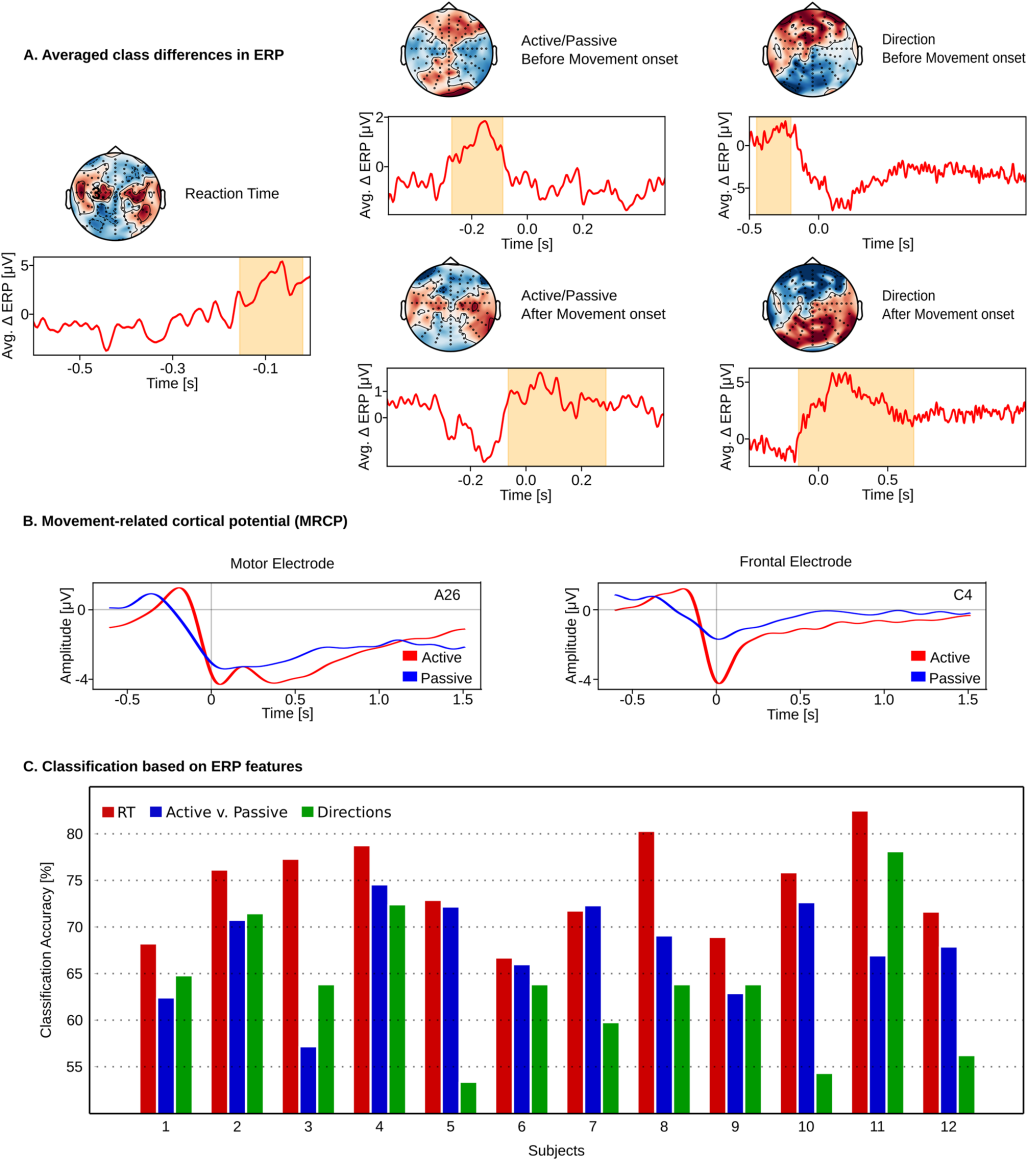
(**A**) Spatiotemporal cluster statistics on the EEG sensors revealed timing and location (channels) in which ERP differences between classes were significant for RT, active vs. passive, and directions. Curves were averaged across all subjects, and the yellow-shaded regions show significant time intervals. (**B**) MRCP from electrodes placed above the motor (A26) and frontal cortex (C4) showing negative potential that started at around 0.25 s before the movement. Curve averaged over all subjects. MRCPs for additional electrodes are shown in Supplementary Material (Fig. S1). (**C**) Leave-one-subject-out accuracies obtained using a logistic regression classifier on the raw EEG epochs for the three classification tasks.

**Figure 3:**
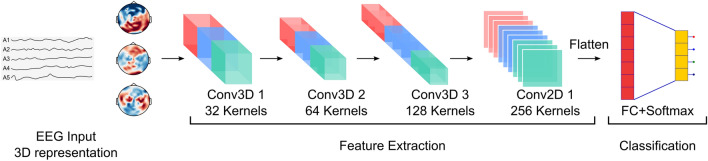
The proposed 3D-CNN architecture. Here, the 5-layer network received topography-preserving 3D EEG inputs and performed convolutions in both sensor and time spaces. Extracted features were fed to the classification layer for class prediction.

**Table 1:** 3D-CNN classification accuracies (%).

Subjects	Leave-one-subject-out			Subject-specific	
	RT	Active/passive	Directions	Active/passive	Directions
1	80.00	71.42	82.85	88.09	85.71
2	79.68	77.61	83.81	95.71	80.95
3	81.03	75.23	72.38	87.65	84.32
4	82.25	82.38	84.76	87.41	90.47
5	79.68	81.90	77.14	96.28	80.95
6	79.51	80.60	72.38	95.13	89.65
7	74.51	86.19	81.20	92.39	84.65
8	80.00	86.67	87.61	93.57	84.32
9	73.91	90.00	83.81	91.12	81.20
10	85.33	82.85	85.71	97.26	92.48
11	81.57	80.47	95.23	88.36	90.45
12	80.26	79.52	77.14	87.45	85.71
Mean	79.81 ± 3.07	81.23 ± 4.87	82.00 ± 6.24	91.70 ± 2.56	85.90 ± 2.17

**Figure 4:**
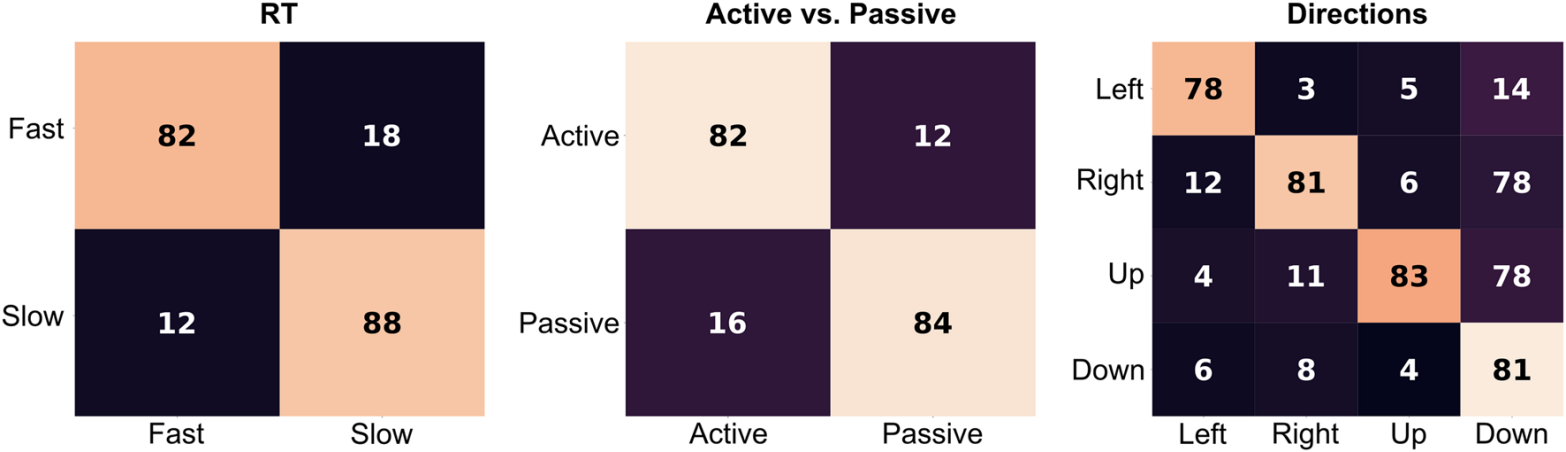
Averaged confusion matrices for the three classification tasks when evaluated using leave-one-out. Numbers are in percentage.

**Figure 5:**
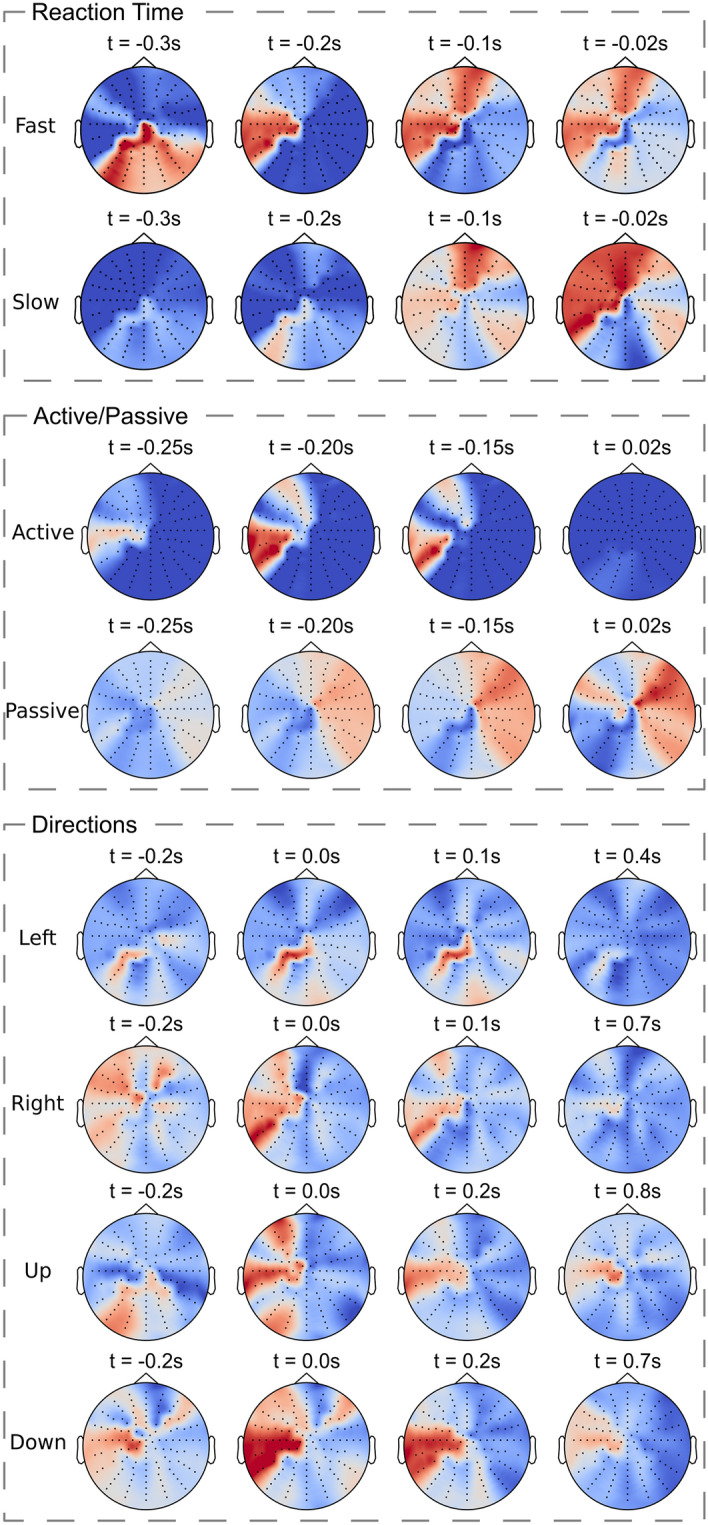
Neurophysiological network interpretation. Gradient-weighted Class activation maps for an individual validation subject for all classification tasks revealed location and timing of activated brain areas. Maps were averaged over all validation trials. The activation maps for all the remaining subjects are provided in the supplementary material (Figs. S2–S12).

**Figure 6:**
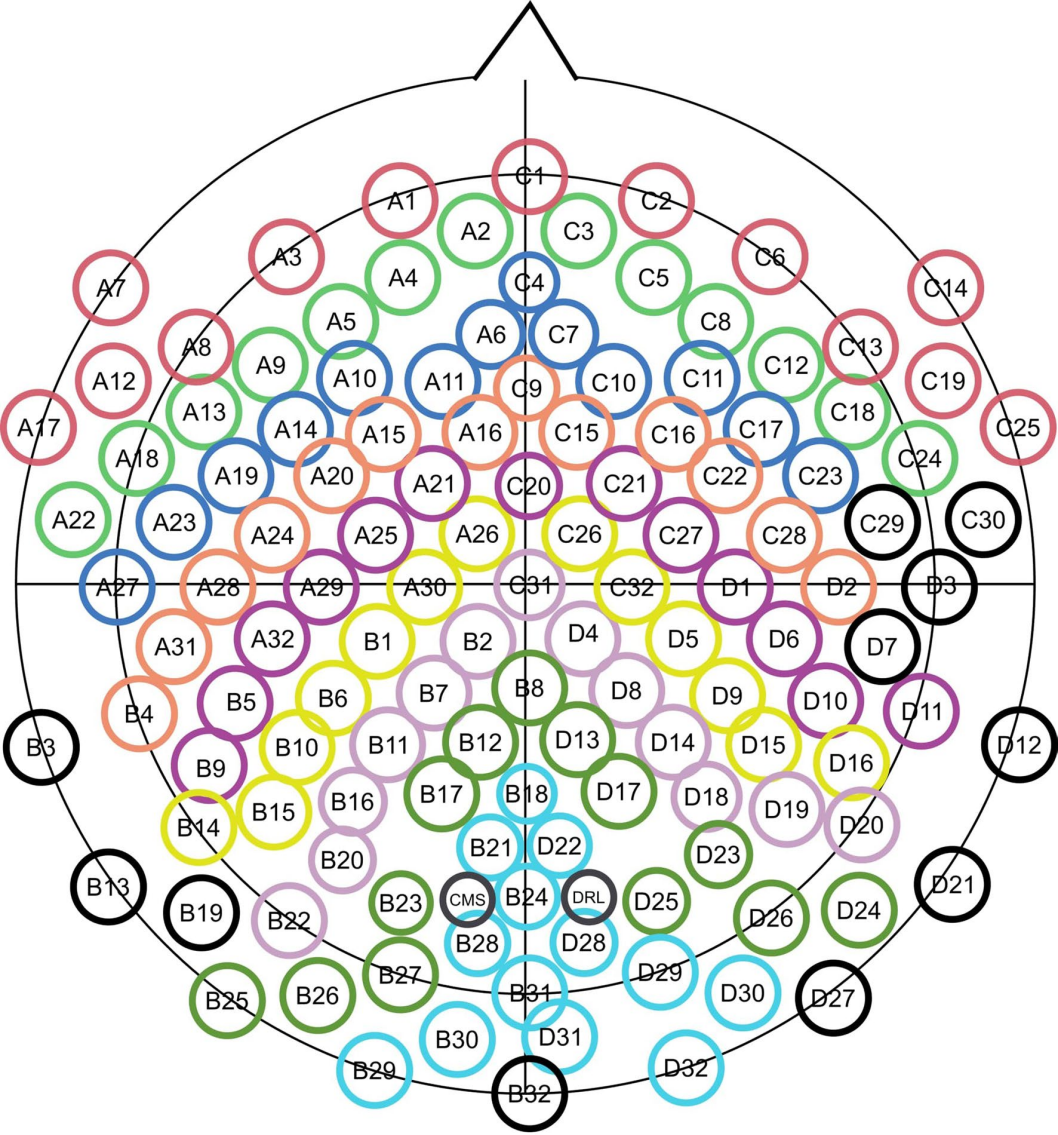
Input representations preserving the brain’s topography applied on BioSemi’s 128 sensor layout. Same colored sensors appeared in the same row in the spatial map. To ensure that the number of sensors per row are same, peripheral channels that were in close proximity to the picked channels were dropped. The dropped sensors are indicated with black outline.

## Results

### Composition of movement components datasets

Data were acquired from an IRB approved in-house experiment where 12 subjects performed goal-directed arm movements on a plane using BioNik’s InMotion Arm Rehabilitation Robot (Fig. 1A). We constructed the following datasets:

1. *RT dataset* We discretized the RT into two classes—slow and fast (see “Methods” section). This dataset consisted of 50 concurrent EEG trials on average per class, for each subject.
2. *Active/passive dataset* We separated the acquired data into active and passive classes, depending on whether there was an assistive force provided by the apparatus. This dataset consisted of 208 EEG trials per class, for each subject.
3. *Directions dataset* We separated the data into 4 classes representing the 4 orthogonal directions—left, right, up, and down movements. This dataset consisted of 52 concurrent EEG trials, per direction, for each subject.

### Class-wise differences in evoked response potential (ERP)

To segment the EEG trials for the 3D-CNN classification, we estimated the time intervals in which the class-wise differences in the EEG signals were statistically significant. To do so, we conducted significance tests on the evoked response potential (ERP) differences between the classes for the three classification tasks using spatio-temporal cluster statistics^34^. We identified clusters of channels and time segments with significant ERP differences (p-value< 0.05) (Fig. 2A). Interestingly, for all the classification tasks, signals prior to the onset of the movement were found to have significant class-discriminatory differences. This is probably related to the presence of movement-related preparatory slow cortical potentials (SCP)^35,36^ that have long been implicated in motor control^37,38^. To confirm this, we computed the movement-related cortical potential (MRCP) by averaging the low-frequency (1–4 Hz) EEG trials for all the subjects (Fig.  2B). We found a negative potential starting at around 0.25s before the movement and ending in a negative peak right after it, gradually increasing to the baseline thereafter. This pattern is consistent with MRCPs observed in goal-directed tasks^39,40^. The initial negative potentials are indicative of motor preparation, with the subsequent rise corresponding to motor execution^41^. The negative peak amplitude was larger for active movements than for passive, also aligning with studies suggesting that more negative peak amplitudes correspond to more complex tasks^36^. The identification of the time intervals allowed us to apply a logistic regression classifier on the raw EEG epochs (Fig.  2C) and build a baseline result for comparing classification accuracies.

### 3D-CNN classification results

To decode the EEG for classifying the complex movement components, we developed a 5-layer 3D-CNN (Fig. 3) with topography-preserving EEG inputs. The network performed 2D convolution in the sensor space and 1D convolution in the temporal space, extracting task-discriminative spatiotemporal features from the EEG data.

We evaluated the 3D-CNN using the following criteria:

1. *Leave-one-subject-out evaluation* To measure the generalization performance of the network, we used the leave-one-subject-out technique ^42^. Our 3D-CNN generalized well across all subjects, achieving average accuracies of $79.81\% \pm 2.37\%$ for RT, $81.23\% \pm 4.87\%$ for active/passive classification, and $82.00\% \pm 6.24\%$ for directions; and outperforming the modern 2D-CNN by 4.6% on RT, 1.1% on active/passive, and 6.74% on direction classification. The 3D-CNN also outperformed the popular LSTM networks by 12.32% on RT and 29.84% on direction classification. In addition, the individual accuracies (Table 1) depict a low variability in the performance despite the high inter-subject variability that exists in the EEG data.
2. *Subject-specific training* To test the ability of the 3D-CNN to infer each individual’s movement components when data from only that person are available, we trained and evaluated our 3D-CNN for each subject separately (Table 1) for datasets that had enough EEG trials per subject. The 3D-CNN exhibited an even higher average accuracy of $91.70\% \pm 2.56\%$ for active/passive and $85.90\% \pm 2.17\%$ for directions. The high accuracies suggest that the network is robust to intra-subject variance.
3. *Training on all data* To test how the 3D-CNN can perform when data from a large pool of subjects is available before-hand, we evaluated the 3D-CNN on data from all subjects, partitioned randomly into train and test in the ratio 4:1. As expected, the 3D-CNN’s performance in this case improved over the leave-one-out evaluation, achieving average accuracies of $84.68\% \pm 3.68\%$ for RT, $87.34\% \pm 2.83\%$ for active/passive classification, and $88.14\% \pm 3.83\%$ for directions.

To test whether the high accuracy is achieved because of a bias in the network classifying a particular class better than the others, we computed the averaged confusion matrices. The performance of 3D-CNN was found to be uniform across classes and datasets (Fig. 4). This was reinforced by the F1 scores for the three tasks:0.88 (RT), 0.89 (active/passive), and 0.91 (directions).

### Network interpretation using class activation maps

To compute the localization maps indicating the location and timing of the activated brain areas that were the most relevant for classification, we developed a gradient-weighted class activation map (Grad-CAM) method (see “Methods” section) for EEG data (Fig.  5):

- *RT* For classifying both slow and fast RT, Grad-CAM identified the most relevant brain locations to be below the EEG sensors covering the contralateral supplementary motor area (SMA), premotor cortex (PMC), primary motor cortex (M1) and orbitofrontal cortex regions. The activation of these brain areas is consistent with several experimental studies on various upper limb movements ^31,33^. Interestingly, these identified regions also correspond to the brain regions that exhibited negative potentials in their MRCPs (Fig.  2B; Fig. S1 in Supplementary Material), which further reinforces their contribution to movement preparation. We identified the important time intervals at around t = − 0.1 s for slow-RT class, while the activation for fast-RT class originated at around t = − 0.3 s, where t = 0 s indicates the start of the movement. This suggests that the important intervals for the slow class were farther in time than the ones for the fast class. The elongated non-decision components of RT in the “slow” class indicated a longer time for stimulus encoding and motor preparation, which aligned with sequential sampling models of the cognitive processes involved in single stage and fast multiple-choice decisions ^43^.
- *Active/passive* For classifying both active and passive classes, the important EEG sensors were found to be the ones above the PMC and M1 regions, also in agreement with recent neurological findings on motor execution of upper limbs^32,44^. The relevant time intervals were before the onset of the motion (t = − 0.2s), suggesting that Grad-CAM captures the timing for motor planning^31^. Interestingly, the activations for the passive class were weaker than that for active class, which was consistent with observations in a recent study differentiating active and passive arm movements^44^.
- *Directions* Similar to active/passive classes, the important EEG sensors identified by Grad-CAM for all four directions were the ones covering the SMA and M1 region, also in accordance with the recent experimental study on upper limb motor execution^32^. Likewise, the relevant time intervals were before the onset of the motion at around t = − 0.2s^31^. As expected^45,46^, the activations exhibited a contralateral pattern, i.e. the brain areas that were identified as salient were opposite to the limb that executed the movement.

## Discussion

In this work, we demonstrated the importance of a biologically relevant 3D-CNN in accurately predicting complex movement components from EEG. The high test accuracies achieved by our 3D-CNN in all the evaluation cases further proved the practical effectiveness of our method in learning the spatio-temporal features existing in the inherently noisy EEG data. Moreover, the correspondence of the learned features with the underlying neurophysiology revealed through Grad-CAM paves the way for introducing an artificial and biological co-learning framework that will spur efforts to enhance functional motor recovery^1,3,4^.

We focused here on the prediction of three movement components—RT, mode of movement: active or passive, and directions. The significance of the prediction of these movement components lies in their role in promoting motor learning and developing neural prostheses. RT is one of the most well-studied behavioral indicators of neurological integrity^47,48^. We have previously shown that RT is responsive to robotic therapy delivered to the ankle of children with cerebral palsy ^3^, confirming its role as a global metric for motor learning. Likewise, the prediction of self-executed movements is important because a significant improvement in motor performance is achieved when training consists solely of voluntary movements^49^. Indeed, the initiation of voluntary movements has been used as a reliable indicator of clinical improvement ^50^. Lastly, functional relevance of the targeted movement indicates that an effective therapy should comprise of training across different movement directions ^51^. Accurate predictions of the three movement components can inform several types of robotic devices on whether the subject wants to initiate a movement or not, the delay (RT) in initiating the movement and the direction of the movement, resulting in a real-time control of the motion.

This work adds to the mounting evidence for the effectiveness of DNNs in predicting cognitive functions from EEG^52^. The proposed 3D-CNN captured in its input representations the spatiotemporal dependencies among the brain areas, and extracted the task-discriminative spatio-temporal EEG features for decoding movement components. While DNNs have exhibited remarkable performance in cognitive domains such as computer vision, natural language processing, and robotics^53,54^, their application in decoding cognitive tasks using EEG has largely been limited to gross classification tasks. Our results suggest that carefully designing the input representations and the network architecture can overcome the inherent variability of EEG signals. In addition, the leave-one-out evaluation suggests that our 3D-CNN generalizes well across subjects, which is promising in being used for new subjects, with minimal to no training. For even better generalization, domain adaptation techniques^55^ can be used to transfer the knowledge learned on one subject to another. Additionally, the ocular artifact removal through ICA can be done online within 1.5 ms^56^; This, alongside an automatic motion detection (either through the kinematics of the apparatus or an external sensor), can enable real-time predictions using our 3D-CNN, with an inference time of less than 52 ms. Such predictions can be used for assessing motor performance and informing the adaptation of the robotic assistance that is currently solely based on kinematics^3^.

For inferences that are reliable in real-world settings and can be extended to other motor components, it is imperative for the network decisions to be interpretable and supported by the domain knowledge. This is crucial as a network may use incorrect features and still achieve a high accuracy on the limited validation set, which typically results in exhibiting unintended behavior when such unreliable systems are deployed in the real-world^29^. We developed a Grad-CAM method for EEG to interpret the decisions taken by the 3D-CNN. The EEG sensors and time segments identified to be relevant to the classification decisions, closely aligned with the current neurophysiological knowledge of the location and timing of activating brain areas for motor execution tasks^31–33,44^. Specifically, the identified brain regions and timing of their activity aligned with reports on M1, PMC, SMA, and orbitofrontal cortex that have been implicated in motor planning^31^ and execution tasks^32,33^. This strong correspondence of the learned features with the underlying neurophysiology increases the reliability of the 3D-CNN to be placed in critical real-time systems for enhancing functional motor recovery.

This work aims to decode, accurately and reliably, the EEG activity associated with well-characterized motor tasks. Our method was developed and evaluated on healthy subjects, which helped us decipher the correlations between normal brain activity and fundamental movement components, which have long been used as robust measures of motor ability^47–49^. For example, RT and active/passive movements have objectively assessed motor performance^3,47^, and have been used to control robots that either imitate limb movement^1^, or attempt to restore its function^3–5,57^. One cannot disregard the intrinsic variability that is present in any neurological disorder that can hinder the generalization of deep learning methods to impaired populations. But with clinical studies on stroke patients now starting to demonstrate the effectiveness of EEG-based BCIs in functional motor recovery^58–62^, the results presented here suggest that developing methods for decoding in real-time movement-related brain activity, is a direction worth pursuing.

## Methods

### Experiment

Twelve naive healthy subjects (age=23 ± 2, 5 females, right handed) participated in this experiment, upon providing informed written consent. The experimental protocol was approved by the Rutgers Institutional Review Board (IRB), and the experiments were then performed in accordance with the relevant guidelines and regulations. The EEG data were recorded using a 128-sensors Biosemi ActiveOne EEG system with a sampling frequency of 1024 Hz. The arm planar movements were performed on the Bionik InMotion rehabilitation arm robot. All data (EEG and movement kinematics) were acquired synchronously. Subjects were seated at a 80cm distance from the screen to allow them to comfortably perform the task without having to move their torso. We developed a visually-guided goal-directed motion task and asked the subjects to perform it on the arm rehabilitation robot. The task environment comprised of a pointer and a target box, similar to ^63^. The pointer indicated the current position of the end-effector of the robotic arm in the 2D plane of motion. Subjects were asked to move the pointer to the target box (active mode), or let the robot guide their arm (in passive mode). The passiveness of the movements was verified by the sensors in the robotic arm that measured the assistive forces, with the passive movements recording assistive forces of greater than 0.8 N. After the pointer entered the target box, the next target box appeared with a delay of 20 ms with an added jitter sampled from a uniform distribution in range [− 10 ms, 10 ms]. The target appeared at random in any of the four orthogonal directions—left, right, up or down. Each subject performed 416 trials (208 active) , where successive movements were separated by at least 2 s, which is enough time for the signals driving the 3D-CNN model to be discriminatory.

### EEG preprocessing and labeling

We preprocessed the EEG data to get rid of the contamination in the EEG data. To remove the low and high frequency artifacts and drifts, we applied a bandpass filter of 0.1–40Hz. We then applied independent component analysis (ICA)^64^ to get rid of ocular artifacts. Subsequently, we segmented the data into trials containing the events of interest. The time window for segmentation was based on the significance test conducted on the ERP differences between the classes (see “Results” section): − 0.5 s to 0 s for RT, − 0.5 s to 0.5 s for active vs. passive, and − 0.5 s to 1.5 s for direction classification (with t = 0 s indicating the start of the motion). Lastly, we normalized the segmented trials using z-score normalization and downsampled all the trials to 250 Hz to reduce the computational load. To compute the RT, we measured the time difference between the onset of a stimulus and the start of the movement, where the movement was said to be started when the velocity exceeded a certain threshold. We discretized the RT into two classes—fast and slow, by choosing suitable thresholds that were determined from the histogram of RTs for each subject separately, based on the distribution of their RT across the experiment. In addition, all trials corresponding to RTs that were outliers, i.e. less that 0.15 s and greater than 0.8 s were removed from consideration^65^.

### Topography-preserving EEG input representation

In representing inputs to the neural network, we preserved important spatial information that exist in the EEG data, which allowed convolution to exploit all the spatial dependencies that exist among brain areas. To do so, we mapped the spatially distributed sensors onto a 2D matrix, which we refer to as spatial map. Each row in the spatial map contained signals from sensors that were immediate neighbors of each other on the sensor layout (Fig. 6). To ensure that the number of sensors in each row were the same, we removed 11 peripheral channels that were in close proximity to the picked channels. This can be done without much loss of information since our high-density EEG system oversamples cortical activity, i.e. a sensor picks up a significant aggregate activity also recorded in the nearby sensors^14^.

### 3D CNN architecture

We developed a multilayered 3D-CNN that received topography-preserving EEG inputs and trained it to learn the three classification tasks. The first 3 layers in the network were 3D convolutional layers with kernel size 3 $\times$ 4 $\times$ 5. The next layer was a 2D convolutional layer with the kernel size 3 $\times$ 5. We added the 2D convolution layer to partially overcome the problem of high number of parameters associated with the 3D CNN. We passed the outputs of each convolutional layer through ReLU non-linearities and then applied batch normalization to normalize the ReLU outputs to zero mean and unit variance. Batch normalization has regularization properties and is helpful in preventing overfitting^66^. We also applied max pooling at the end of each layer to reduce computational load. Max pooling has also the desirable property of translational invariance which relates to better generalization across subjects. The last layer was a fully connected layer with softmax that took in the flattened feature vector produced by the last convolutional layer and converted it to class probabilities. The choice of the CNN hyper-parameters, i.e. the number of layers, kernel size, etc. were limited by the training data size and the input dimension, and were found using a grid search over the allowable hyper-parameters space.

### Training details

For each subject *i*, we created a dataset $D^i = \{(X_i^1, y_i^1), (X_i^2, y_i^2),\ldots ,(X_i^{n_i}, y_i^{n_i})\}$, where $n_i$ denotes the number of trials recorded for that subject. For every trial *j*, $X^j \in \mathbb {R}^{13 \times 9 \times T}$ is a 3 dimensional matrix denoting the topography-preserving EEG inputs. *T* is the duration of recording of each trial. The labels $y^j$ of the trial *j* contains a value from $\{0,\ldots ,K\}$ where *K* is the number of classes for the classification problem. The neural network computes a mapping from the EEG trial to the labels, $f(X^j, \theta ):\mathbb {R}^{13 \times 9 \times T}\rightarrow \mathbb {R}^{K}$ where $\theta$ are the trainable parameters of the network. The network is trained to minimize the average loss over all training examples:

1 $$\begin{aligned} \hat{\theta } = \hbox {arg}\,\hbox {min} \frac{1}{N} \Sigma _{i=1}^N l\left( X^i, y^i;\theta \right) , \end{aligned}$$

where *N* denotes the number of training examples and *l* is the loss function which in our case is the cross entropy loss function. We set the batch size to be 64. We used a variant of stochastic gradient descent—Adam^67^ for optimization with learning rate of $10^{-3}$. All networks were trained for 60 epochs.

### Network validation

We evaluated our proposed 3D-CNN in three ways:

1. *Leave-one-subject-out* Data from all but one subject were used for training. Evaluation was done on the left-out subject. This tested the 3D-CNN’s ability to generalize to new subjects, that were not included in training.
2. *Subject-specific training* Data from a single subject were split randomly into training and test in the ratio 4:1. Validation was done on the test data. This tested the ability of the 3D-CNN to predict movement components when trained on individual subjects.
3. *All data* Data from all subjects were split randomly into training and test in the ratio 4:1. This allowed us to determine how well the 3D-CNN performs if it has access to data from a large number of subjects. We trained 10 networks corresponding to 10 such random partitions for each classification task and show the averaged results in the “Results” section.

### Gradient-weighted class activation maps (Grad-CAM)

Grad-CAM is a technique that is very popular in the vision community to compute the saliency maps for classification decisions^30^. Specifically, Grad-CAM produces localization maps which highlight the pixels in the input image that are important for its classification. To interpret our 3D-CNN using Grad-CAM, we first reduced the maxpool window size to obtain higher resolution EEG feature map as the output of the last 2D-convolutional layer. We then computed the gradients of the score of the predicted class with respect to the EEG feature maps, and averaged them in space and time to obtain importance scores. A weighted combination of the importance scores and feature maps produced coarse activation maps which were then upsampled to the size of EEG inputs to produce high-resolution class activation maps. These class activation maps were generated for validation subjects with networks trained using leave-one-out validation.

### Ethics approval and consent to participate

The experimental protocol was approved by the Rutgers Institutional Review Board (IRB), and the experiments were then performed in accordance with the relevant guidelines and regulations. All subjects provided informed written consent.

## Supplementary Information

Supplementary Information.

## Data Availability

The datasets generated during the current study are available from the corresponding author on reasonable request.
