# Supplementary material for: A neurophysiologically interpretable deep neural network predicts complex movement components from brain activity
Source: Sci Rep. 2022 Jan 20;12:1101. doi: 10.1038/s41598-022-05079-0 (PMC8776813; doi:10.1038/s41598-022-05079-0)
Supplement: Supplementary file 1 — Supplementary Information. [file 41598_2022_5079_MOESM1_ESM.pdf]

A Neurophysiologically Interpretable Deep Neural Network Predicts Complex Movement Components from Brain Activity: Supplementary Materials

Neelesh Kumar[1] **and Konstantinos P. Michmizos**[1,*]

[1]Computational Brain Lab, Department of Computer Science, Rutgers University, NJ, USA
[*]michmizos@cs.rutgers.edu

2D-CNN Classification Results

The multilayered 2D-CNN received preprocessed EEG trials acquired from 128 channels and sampled at 250 Hz. The first 4 layers in the network were 2D convolutional layers with kernel size 3x5 and 32, 64, 128, and 256 channels respectively. We passed the outputs of each convolutional layer through ReLU non-linearities and then applied batch normalization to normalize the ReLU outputs to zero mean and unit variance. We also applied max pooling at the end of each layer to reduce computational load. The last layer was a fully connected layer with softmax that took in the flattened feature vector produced by the last convolutional layer and converted it to class probabilities. The choice of the CNN hyper-parameters, i.e. the number of layers, kernel size, etc. were limited by the training data size and the input dimension, and were found using a grid search over the allowable hyper-parameters space. The leave-one-subject-out accuracies for the 2D-CNN for each classification tasks are shown in Table 1.

CNN-LSTM Classification Results

The multilayered CNN-LSTM received preprocessed EEG trials acquired from 128 channels and sampled at 250 Hz. The first 3 layers in the network were 2D convolutional layers with kernel size 3x3 and number of channels being 16, 32, and 64 respectively. We passed the outputs of each convolutional layer through ReLU non-linearities and then applied batch normalization to normalize the ReLU outputs to zero mean and unit variance. We also applied max pooling at the end of each layer to reduce computational load. The flattened feature vector produced by the last convolutional layer was sent to an LSTM layer with 32 hidden units. Finally, the LSTM outputs were fed to a fully connected layer with softmax that converted it to class probabilities. The leave-one-subject-out accuracies for the CNN-LSTM are shown in Table 2.

Additional Results

We show MRCPs for additional electrodes in Fig. S1. We also provide here the activation maps for all the subjects as an indicator of group results (Figs. S2-S12).

Subjects	Leave-one-out		
	**RT**	**Active/Passive**	**Directions**
1	67.30	75.67	82.54
2	65.85	83.32	76.19
3	69.64	82.31	69.52
4	81.96	79.10	82.85
5	73.21	75.16	74.28
6	75.00	77.20	65.71
7	77.58	74.19	74.49
8	79.31	80.00	82.85
9	76.78	88.20	64.76
10	72.41	92.12	75.28
11	82.35	76.25	93.33
12	80.32	79.12	73.33
**Mean**	**75.14 ± 5.32**	**80.13 ± 5.26**	**76.26 ± 7.76**

Table 1. 2D-CNN Classification Accuracies (%)

Subjects	Leave-one-out	
	**RT**	**Directions**
1	67.85	53.33
2	70.58	70.47
3	54.68	56.19
4	70.58	54.28
5	69.41	46.66
6	70.58	49.52
7	65.95	51.67
8	67.21	70.47
9	64.06	42.85
10	74.60	30.47
11	60.93	57.14
12	73.43	52.16
**Mean**	**67.49 ± 5.32**	**52.16 ± 10.42**

Table 2. CNN-LSTM Classification Accuracies (%)

Movement-related cortical potential (MRCP)

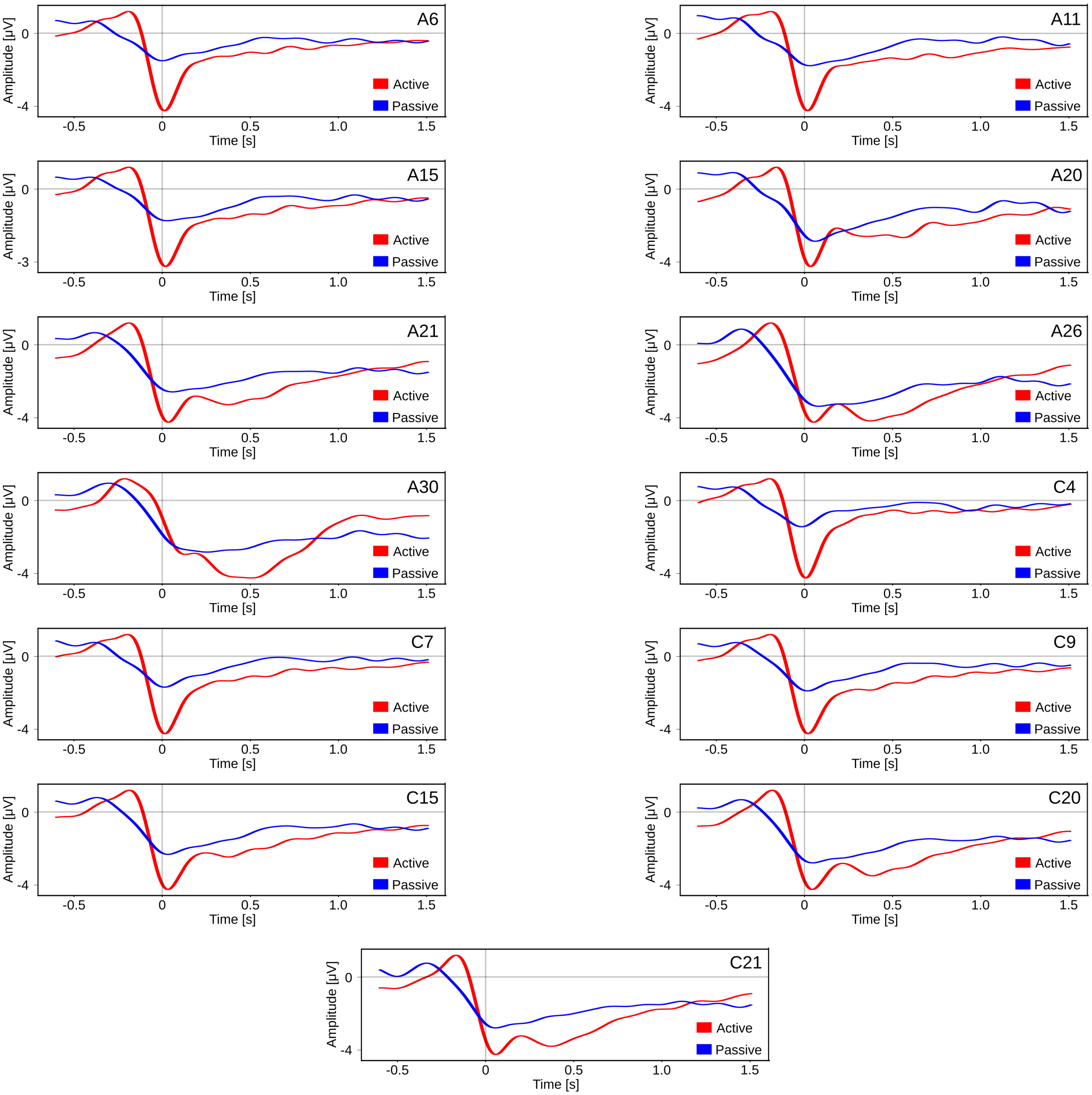

Figure S1. MRCPs for additional electrodes

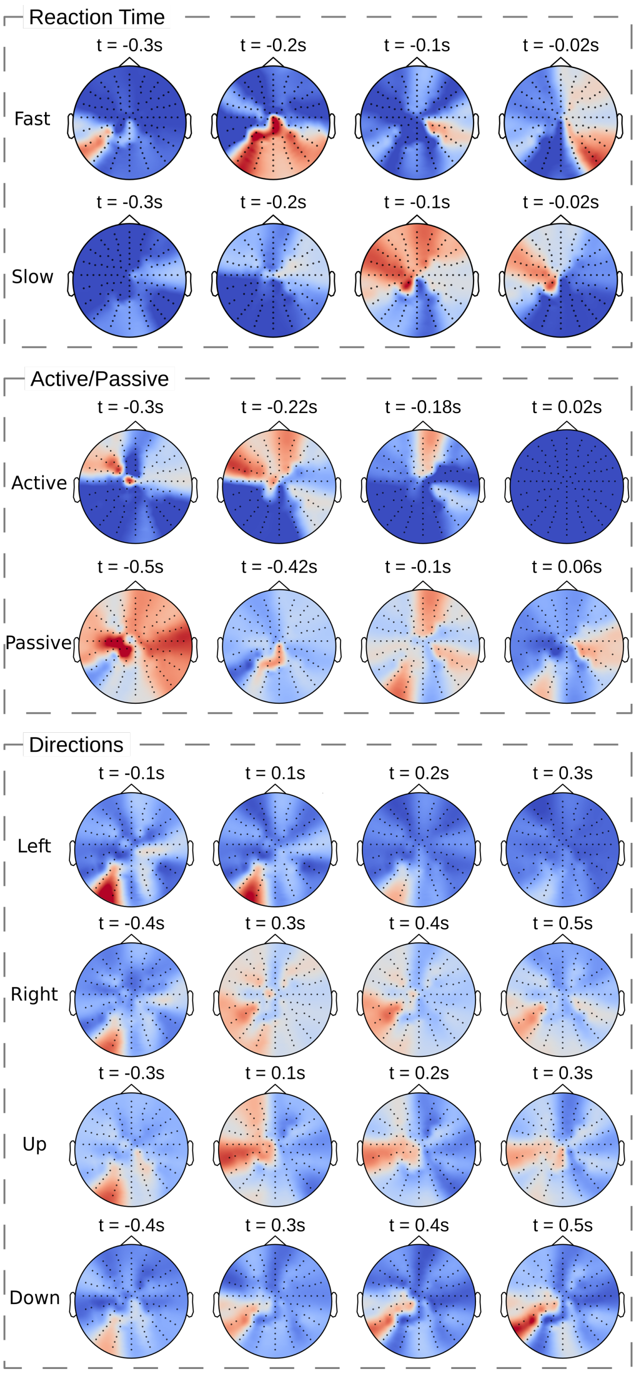

Figure S2. Activation maps for Subject 2

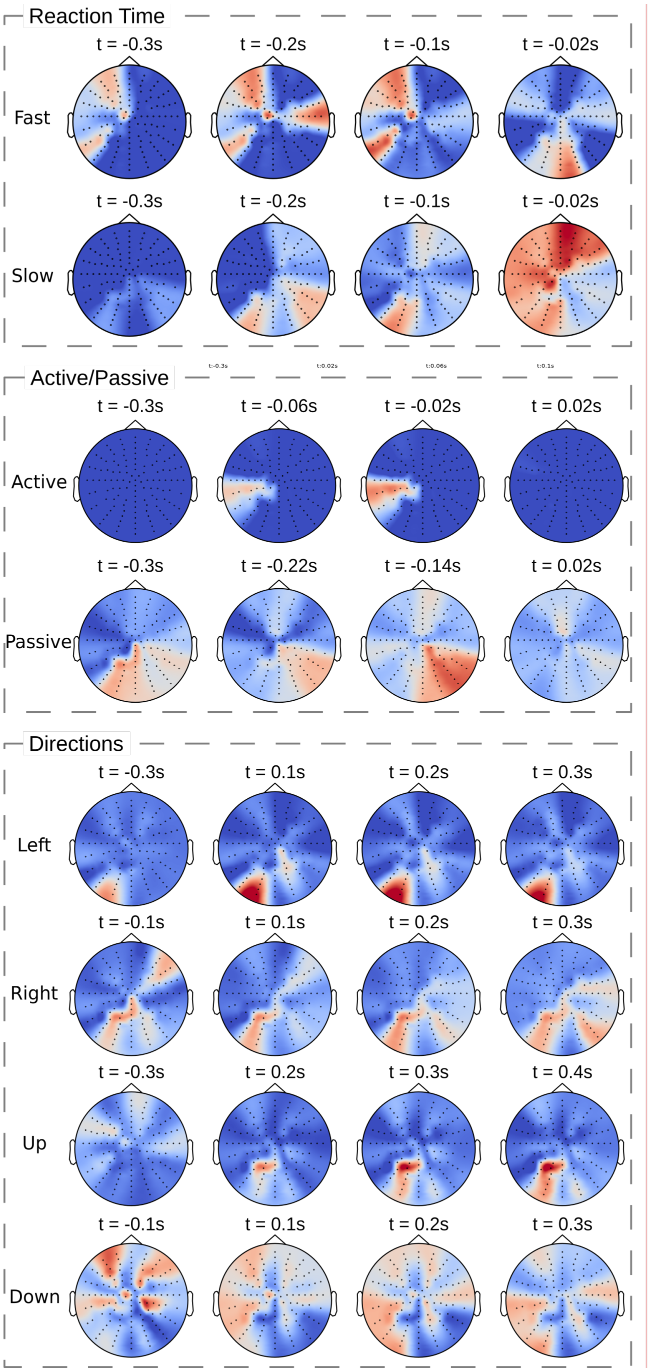

Figure S3. Activation maps for Subject 3

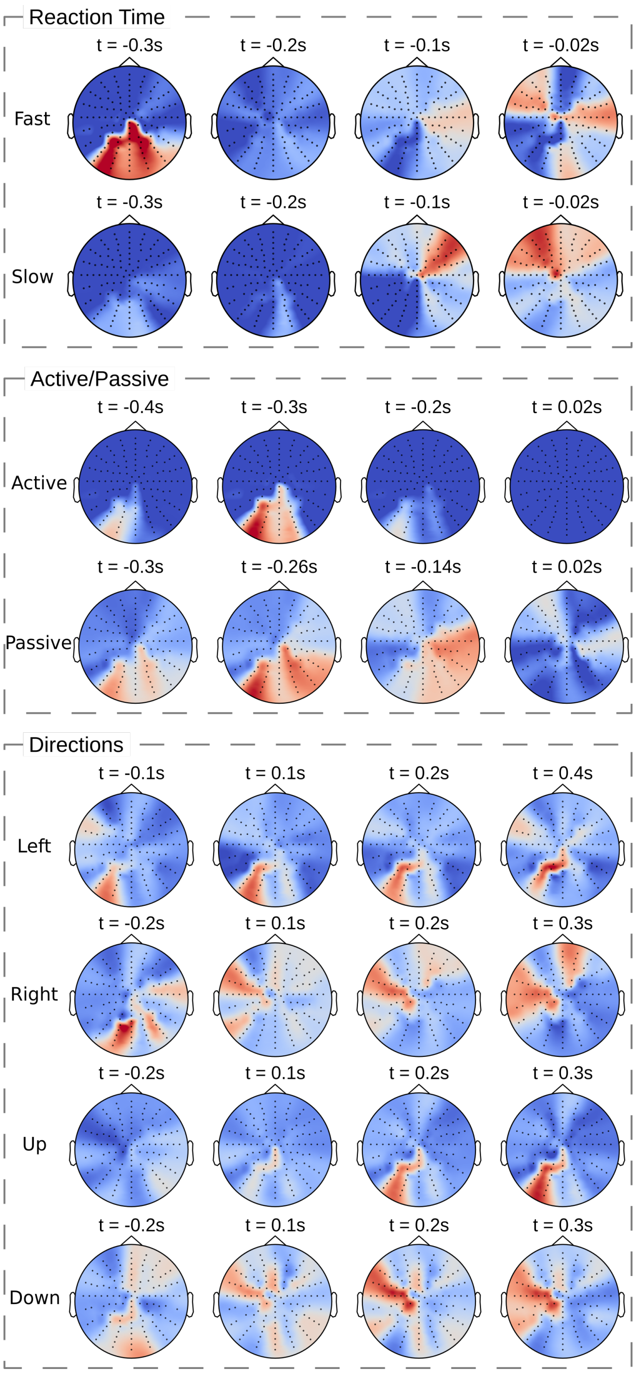

Figure S4. Activation maps for Subject 4

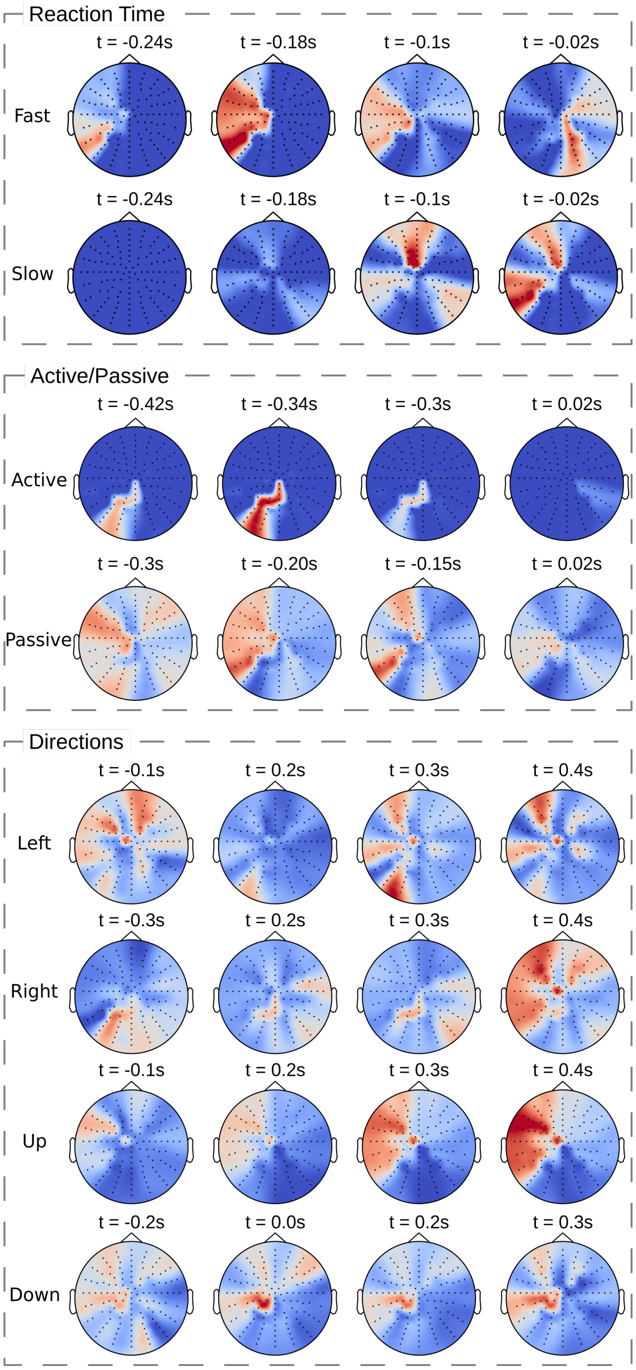

Figure S5. Activation maps for Subject 5

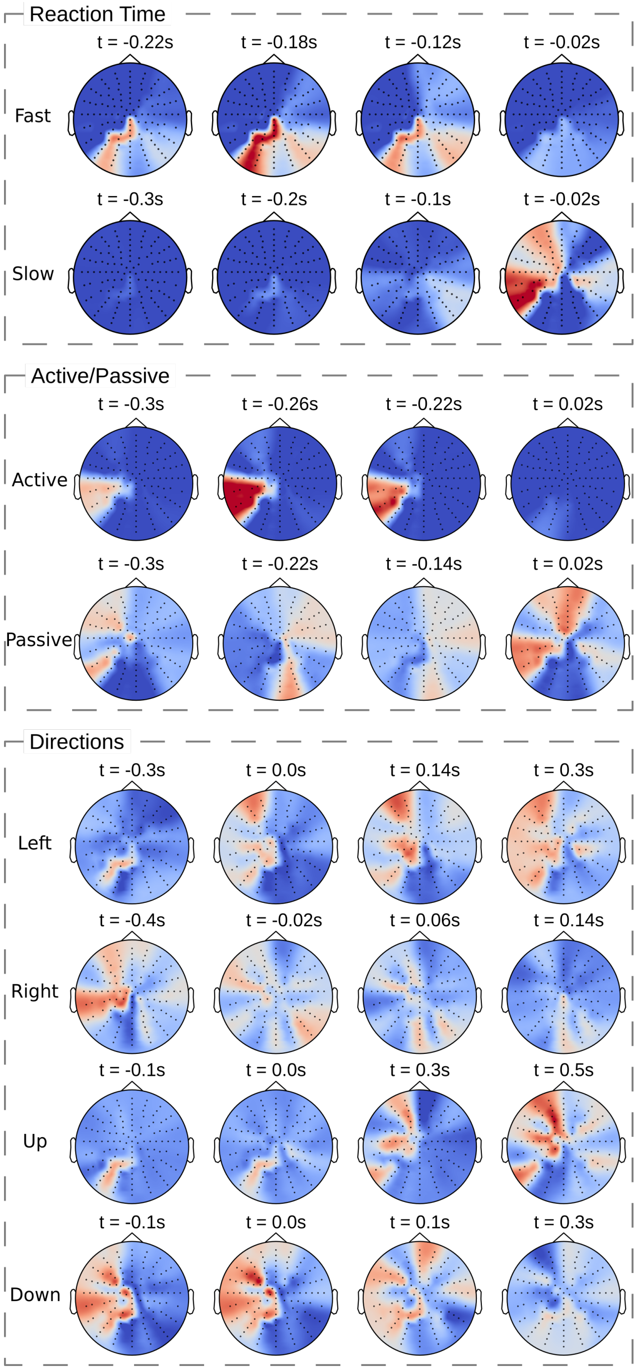

Figure S6. Activation maps for Subject 6

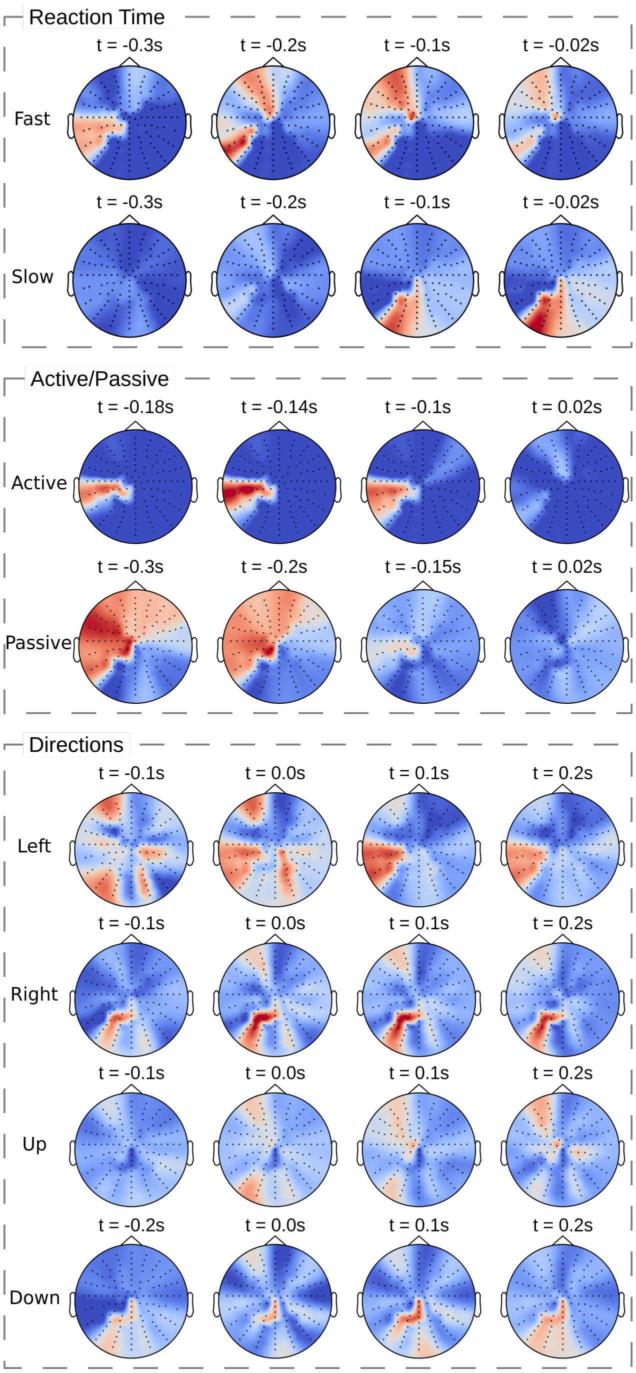

Figure S7. Activation maps for Subject 7

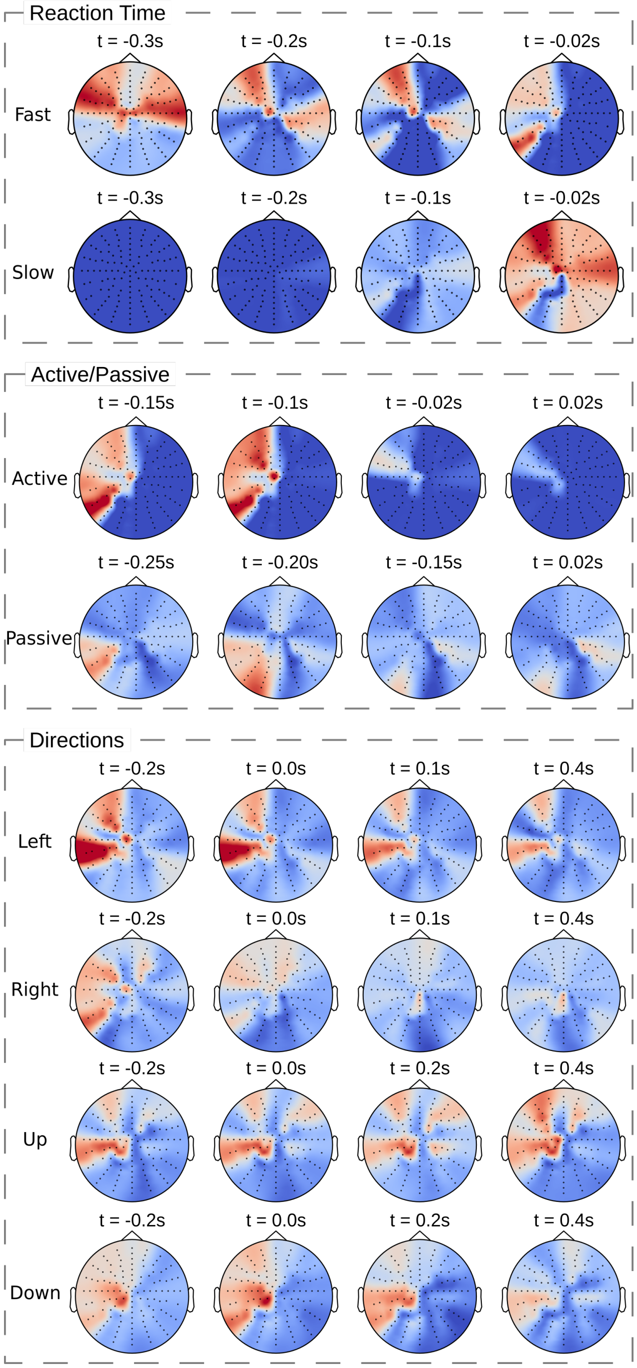

Figure S8. Activation maps for Subject 8

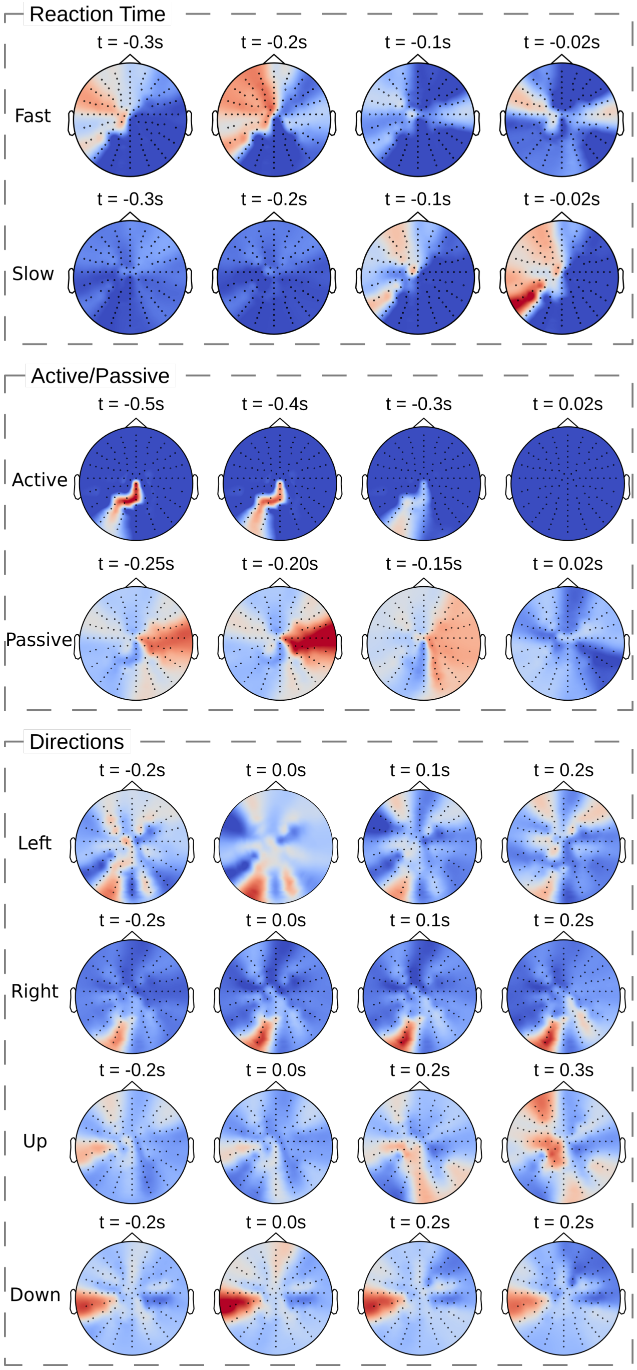

Figure S9. Activation maps for Subject 9

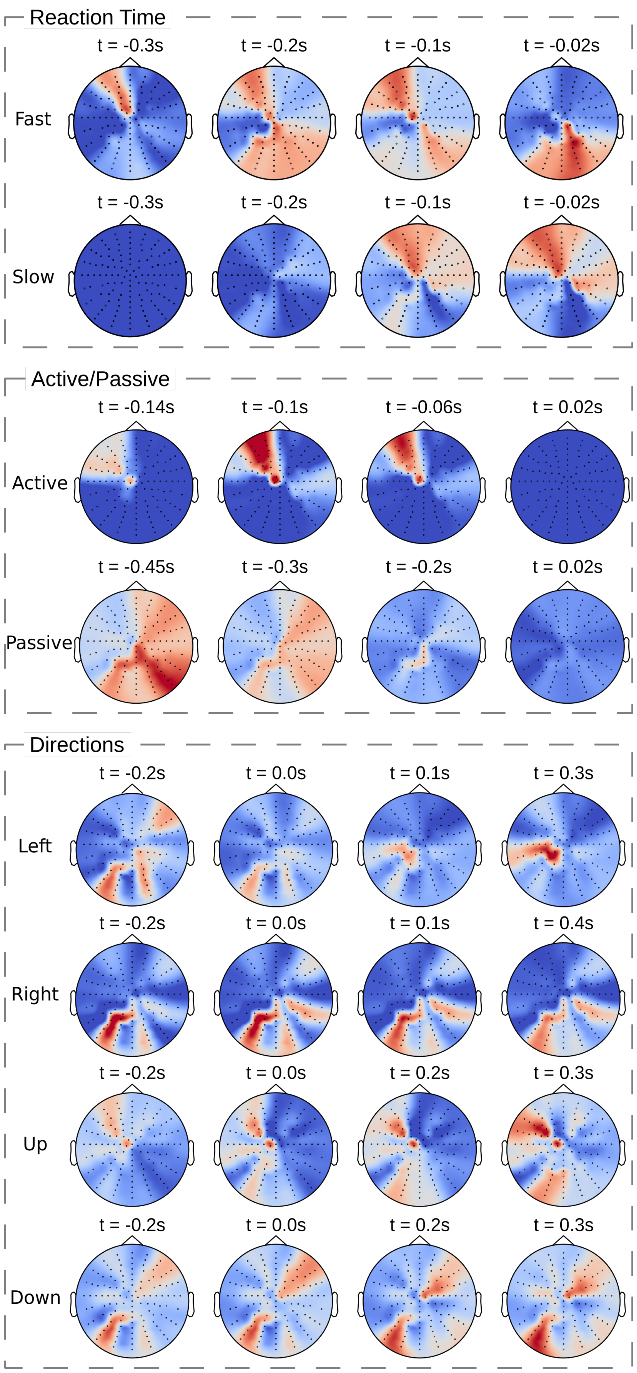

Figure S10. Activation maps for Subject 10

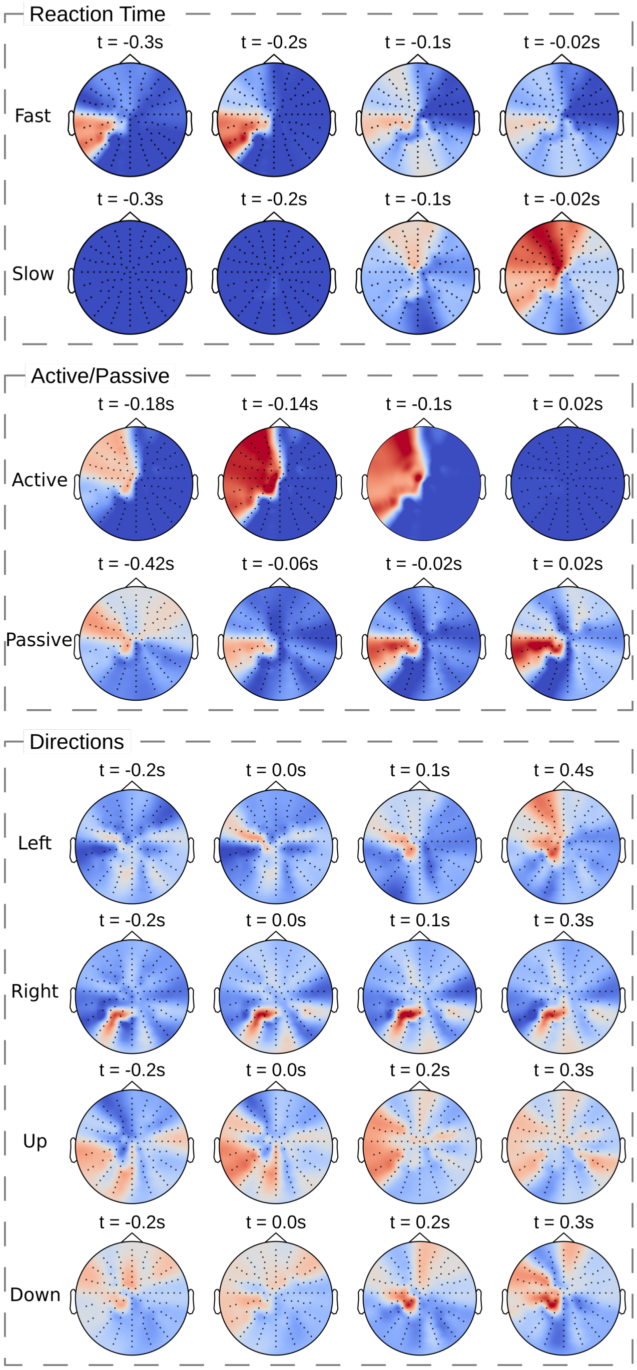

Figure S11. Activation maps for Subject 11

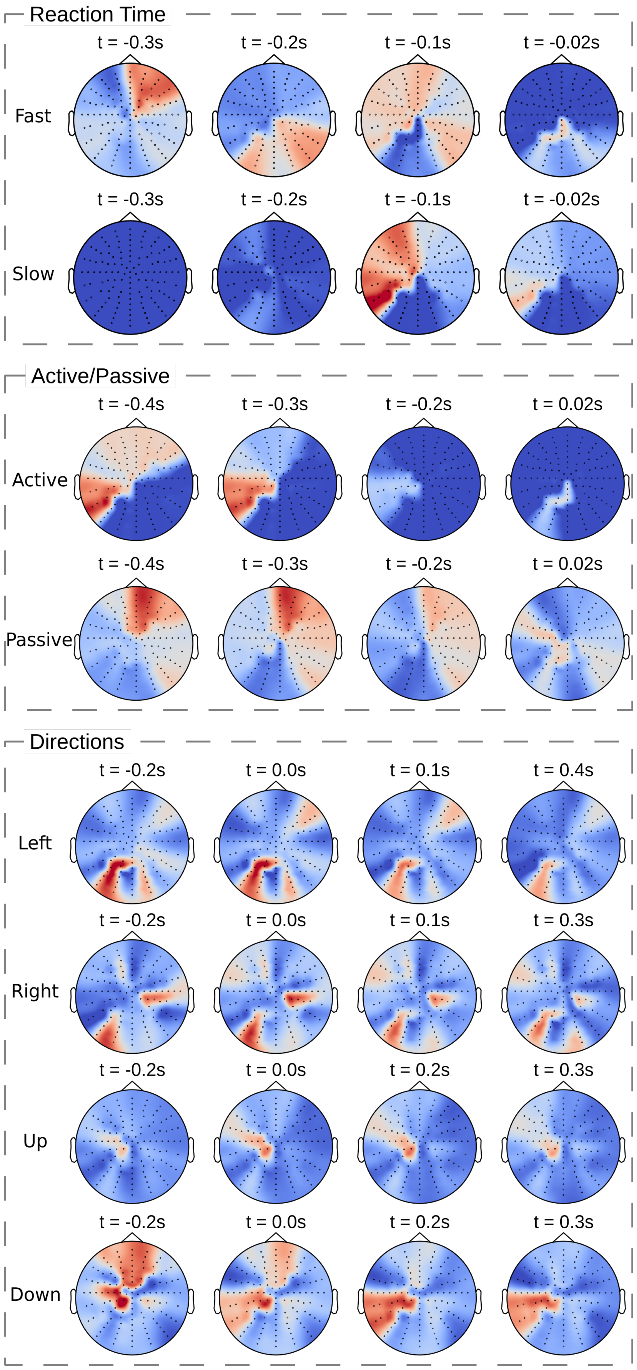

Figure S12. Activation maps for Subject 12
